# Certificates of the Cause of Death

**Published:** 1898-03-26

**Authors:** 


### The Hospital

A Journal of

The Medical Sciences and Hospital Administration.

Vol. XXIII.—No. 600.] Edited by Sir Henry Burdett, K.C.B., and Solomon C. Smith, M.D., M.R.C.P. [March 26, 1898.

### Certificates of the Cause of Death.

The police reports of Tuesday contained an account of a curious case, we believe the first of its kind, in which a registered medical practitioner was summoned for refusal to give a certificate of the cause of death. The defendant was Mr. Patrick Lyons Blewitt, of Barking Road, and he was summoned at the instance of Mr. Hilleary, the Coroner for West Ham. It appeared that Mr. Blewitt attended a Mrs. Desborough, who, presumably after her confinement, was sent to an asylum, and that he also attended her baby, and saw it on the day before its death, which occurred when it was two months old. His charges, which amounted to twelve shillings, had not been paid, and, when the father of the child applied to him for a certificate, he refused to give it until they were. The father went to ask for the certificate on seven different occasions, but he presumably went empty handed, and the coroner, eight days after the death, held an inquest, at which Mr. Blewitt was called upon to give evidence. He said that the child died of syncope, and that it had been suffering from debility from its birth. The magistrate, Mr. Ernest Baggallay, said that the prosecution had been very properly instituted, that it was the duty of a medical practitioner who attended a last illness to give a certificate of the cause of death, and that the law would be a dead letter if this certificate could be withheld because a few shillings were due to him. The public were put to the expense of an inquest, for which Mr. Blewitt himself would receive a guinea. Mr. Blewitt had abundant knowledge of the cause of death, and had no excuse for not giving the certificate. He would be fined 40s. and costs, £5 9s. 6d. in all, distress, or twenty-one days.

It is recorded of a Scotch judge that he once sentenced a prisoner with the preliminary remark that the offence of which he had been found guilty was not only eminently displeasing in the sight of the Most High, but that it was also in direct contravention of an Act of Parliament. Mr. Baggallay could not, we think, have quoted more than the last clause of this pronouncement. There is something squalid, it cannot be gainsaid, in the struggle for the few shillings over the corpse of the poor child; but there is something worse even than squalor, there is downright injustice in the law which provides that the registration of the causes of death, in this country, should be maintained in a state of correctness and efficiency at the cost of the medical profession, and that they should be compelled to do this important work without remuneration, even in the case of persons who have never paid them a shilling. So far as we are aware, the case of Mr. Blewitt is the first of the kind which has been brought into court, and it can hardly be doubted that he thought he was within his rights in refusing to certify. The law provides no penalty for his offence; but it is a misdemeanour to refuse obedience to an Act of Parliament, and we presume that the conviction and penalty must have rested upon this ground. How the Coroner was introduced into the business does not appear; because it is a common matter of complaint that a Registrar of Deaths, or his official superior, the Registrar General, has no power to compel people to employ registered medical practitioners, or to prevent them from employing unqualified ones. When a death occurs in a case that has not been attended by a registered practitioner, a statement, from the unregistered—perhaps unqualified—attendant (if there have been any) is usually brought to the Registrar by the informant of the death. The Registrar then has to consider whether the case is one of which the Coroner should be notified; and it is probable that the majority of deaths of infants two months old, who had been deprived of their mothers, and exposed to all the disadvantages of extreme poverty, would hardly be thought to require such a proceeding. Either the Registrar for the Barking Road district must be unusually careful and conscientious, or the Coroner for West Ham must be more particular than many of his learned brethren. There can be no doubt that the prevailing laxity in the registration of deaths is a great evil; but we cannot think it otherwise than hard upon Mr. Blewitt that, the world being doubtless out of joint in this particular, the stress of submitting to the injustice needed as a stimulus to reform should have fallen upon him. It has long been felt by all who are conversant with the working of the Registration Act that its amendment is in many respects very much to be desired, and in no respect more than with reference to the downright dishonesty which now compels doctors to place, without remuneration, their special knowledge at the service of the State. There is no other profession upon which the Legislature would even have attempted to levy such an iniquitous tax, and there is no other which, for sixty years, would have endured it without strenuous efforts to obtain its removal. Submission to gross injustice does not pay, and the best efforts of the profession should be directed towards obtaining release from a burden which never ought to have been imposed upon them.

